# miR-10c Targets *dgat2* and Affects the Expression of Genes Involved in Fatty Acid and Triglyceride Metabolism in *Oreochromis niloticus* Under Heat Stress

**DOI:** 10.3390/ijms26199717

**Published:** 2025-10-06

**Authors:** Wen Wang, Wenjing Tao, Jixiang Hua, Siqi Lu, Yalun Dong, Jun Qiang, Yifan Tao

**Affiliations:** 1 Wuxi Fisheries College, Nanjing Agricultural University, Wuxi 214081, China; linda1995king@163.com (W.W.);; 2 Key Laboratory of Freshwater Fisheries and Germplasm Resources Utilization, Ministry of Agriculture and Rural Affairs, Freshwater Fisheries Research Center, Chinese Academy of Fishery Sciences, Wuxi 214081, China; 3 Key Laboratory of Freshwater Fish Reproduction and Development (Ministry of Education), Key Laboratory of Aquatic Science of Chongqing, School of Life Sciences, Southwest University, Chongqing 400715, China

**Keywords:** *Oreochromis niloticus*, miR-10c, *dgat2*, heat stress, lipid metabolism

## Abstract

Heat stress induces metabolic adaptations in fish, including the regulation of triglyceride (TG) synthesis/degradation to preserve cellular lipid balance and energy homeostasis. Diacylglycerol acyltransferase (DGAT) catalyzes the final step in TG synthesis. However, the molecular mechanisms by which DGAT regulates TG metabolism in heat-stressed fish remain unexplored. Our previous study suggested that miR-10c regulates *dgat2* expression in genetically improved farmed tilapia (GIFT, *Oreochromis niloticus*) under heat stress. Here, we characterized the GIFT miR-10c precursor as a 65-nucleotide transcript yielding a 22 nt mature miRNA (oni-miR-10c). A phylogenetic analysis revealed a high level of miR-10c sequence conservation across species. A dual-luciferase reporter assay confirmed *dgat2* as a direct target of miR-10c. Overexpression of miR-10c in vivo down-regulated *dgat2* transcripts and DGAT2 protein. SiRNA-knockdown of *dgat2* resulted in upregulation of *cpt1α*, *fas*, and *lpl* and downregulation of *hsl*, thereby reprogramming lipid metabolism in GIFT hepatocytes. Thus, the miR-10c-*dgat2* regulatory axis facilitates TG hydrolysis and promotes fatty acid metabolism under heat stress. Our findings highlight miR-10c’s potential as a *dgat2* inhibitor and its function in regulating lipid metabolism in heat-stressed GIFT. Our study reveals a key molecular pathway mediating thermal adaptation of energy metabolism in fish, providing novel targets for preventing heat-induced metabolic disorders.

## 1. Introduction

Many environmental conditions can limit an organism’s physiological processes, including growth and reproduction. Whether they are raised naturally or artificially, fish are unavoidably exposed to a number of stressors, including non-optimal dissolved oxygen and salinity levels, density, and temperature [1]. Among these, temperature affects fish throughout their life cycle and is an essential factor impacting their daily activity [2]. Temperature increases and extremely high summer temperatures have become more common and severe in recent years. Climate change poses a risk to aquaculture, endangering the availability of food for humans [3]. Most fish are susceptible to heat shock, and can suffer physiological damage from which they never recover, or even die, because they are unable to adapt to rising external temperatures [4].

Within a certain range of temperatures, fish undergo physiological, biochemical, and metabolic changes as a result of heat stress. These changes are reflected by increases in the rates of respiration, oxygen consumption, and ammonia and carbon dioxide excretion [5]. The reprogramming of energy metabolism pathways is one of the adaptive mechanisms that functions in fish under heat stress to maintain critical physiological functions and preserve internal homeostasis [6]. Enhancement of lipid metabolism is one way that fish adapt to high temperature stress. Triglycerides (TGs) provide energy and are essential for metabolic homeostasis because they are the primary energy storage molecule in fish [7]. For example, pikeperch (*Sander lucioperca*) exhibited a marked decrease in serum TG levels as the water temperature gradually increased [8], and similar results have been reported for largemouth bass (*Micropterus salmoides*) [9] and Tibetan naked carp (*Gymnocypris przewalskii*) [10]. These changes in TG levels may reflect a physiological regulatory mechanism in fish whereby lipid synthesis and storage are reduced in response to heat stress.

Diglycerides and the fatty acid acyl CoA are covalently bound to produce TGs in a reaction catalyzed by diacylglycerol acyltransferase (DGAT)—this is the last step in TG synthesis. The expression level of DGAT is directly related to TG accumulation in mammals [11]. The two main types of DGATs in animals are encoded by the genes *dgat1* and *dgat2*. *dgat1* is widely expressed in various tissues and organs of animals, and its transcript level is coupled with the metabolic effects of TGs. Overexpression of *dgat1* in mouse adipose tissue and skeletal muscle was shown to significantly increase the flux of diglycerides into the TG synthesis pathway, thereby increasing TG synthesis [12,13]. DGAT2 is crucial for the synthesis and storage of TGs. Knockout of *dgat2* in mice reduced their adipose mass and markedly decreased the TG content [14]. In previous studies, the TG biosynthetic capacity of teleost fish was assessed by determining the *dgat* transcript levels [15,16]. The hybrid offspring of Nile tilapia (*Oreochromis niloticus*) and blue tilapia (*Oreochromis aureus*) exhibit elevated hepatic TG accumulation, concomitant with significantly upregulated *dgat2* mRNA levels and DGAT2 protein expression. This suggests that DGAT2 plays a crucial role in lipid synthesis and metabolism in hybrid tilapia [17]. However, it is still necessary to clarify the precise role of DGAT2 in tilapia’s metabolic response to heat stress.

MicroRNAs (miRNAs) regulate gene expression at the post-transcriptional level through sequence-specific base pairing with target mRNAs, resulting in either transcript degradation or translational repression [18]. miRNAs play a critical regulatory role in the lipid metabolic response to heat stress in both mammals and fish. For instance, in swine (*Sus scrofa*), heat stress was found to impair skeletal muscle glucose and lipid metabolism by modulating 58 differentially expressed miRNAs (30 downregulated, 28 upregulated) and their target genes [19]. Similarly, transcriptome analyses have identified multiple critical metabolic-responsive miRNAs in heat-stressed spotted sea bass (*Lateolabrax maculatus*) [20] and largemouth bass [21]. miR-10 family members exhibit transcriptional co-expression with multiple *Hox* genes and repress the translation of *Hox* transcripts [22]. As a key member of the miR-10 family, miR-10c was found to potentially modulate amino acid and lipid utilization in the skeletal muscle of Chinese perch (*Siniperca chuatsi*) during nutritional restriction and refeeding [23]. Genetically improved farmed tilapia (GIFT, *Oreochromis niloticus*) is an economically important freshwater fish that is mainly farmed in the southern provinces of China. Elevated summer temperatures limit the development of the GIFT aquaculture industry. In our previous study, we observed that miR-10c transcript levels significantly increased under hyperthermic conditions, inversely correlating with *dgat2* mRNA levels [24]. This suggested that the overexpression of miR-10c might be a key mechanism for the rapid adaptation of lipid metabolism in GIFT under hyperthermia.

In this study, therefore, we first verified that *dgat2* is a potential target of miR-10c. Building upon this finding, we determined the effect of *dgat2* knockdown by siRNA on the TG content and lipid metabolic pathways in GIFT hepatocytes. We then investigated the regulatory mechanisms through which miR-10c overexpression governs hepatic lipid metabolism in GIFT under heat stress. Our findings provide a theoretical foundation for in-depth research on the molecular regulatory mechanisms and adaptive strategies of fish under heat stress.

## 2. Results

### 2.1. Sequence Characterization of miR-10c

**Figure 1:**
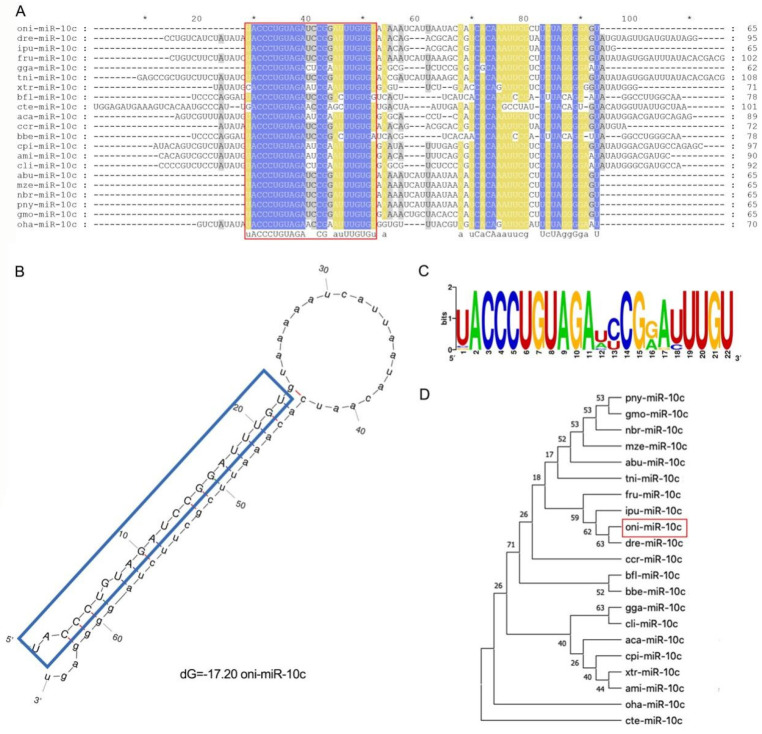
Bioinformatic analysis of miR-10c. (**A**) Cross-species alignment of miR-10c precursor sequences downloaded from miRBase. The red boxes indicates the distribution of the mature form of miR-10c within the precursor sequence for each species; (**B**) Predicted secondary structure of oni-miR-10c precursor. Blue box represents the mature sequence of oni-miR-10c; (**C**) Conservation analysis of mature miR-10c sequences; (**D**) Phylogenetic tree of mature miR-10c sequences from different species, reconstructed using the neighbor-joining method in MEGA 11.0. Note: * represents conserved site.

First, we characterized the GIFT miR-10c precursor as a 65 nt transcript yielding a 22 nt mature miRNA (oni-miR-10c). The complete sequence was as follows: (mature sequence underlined): UACCCUGUAGAUCCGGAUUUGUGUAAAAAUCAUUAAUACAAUCACAAAUUCGCUUCUAGGGGAGU. miR-10c sequences from multiple species were obtained from the miRBase database and aligned using MEGA software (Figure 1A). The predicted secondary structure of the oni-miR-10c precursor showed the characteristic stem-loop fold with high structural stability (Figure 1B). Conservation analysis revealed high sequence conservation among mature miR-10c sequences, with the seed region (5′-ACCCUG-3′) being completely identical across all examined species (Figure 1C). A phylogenetic tree was constructed using the neighbor-joining method. In the tree, oni-miR-10c clustered with other fish orthologs in a distinct clade (Figure 1D), consistent with their evolutionary relationships.

### 2.2. Verification of dgat2 as a Target of miR-10c

**Figure 2:**
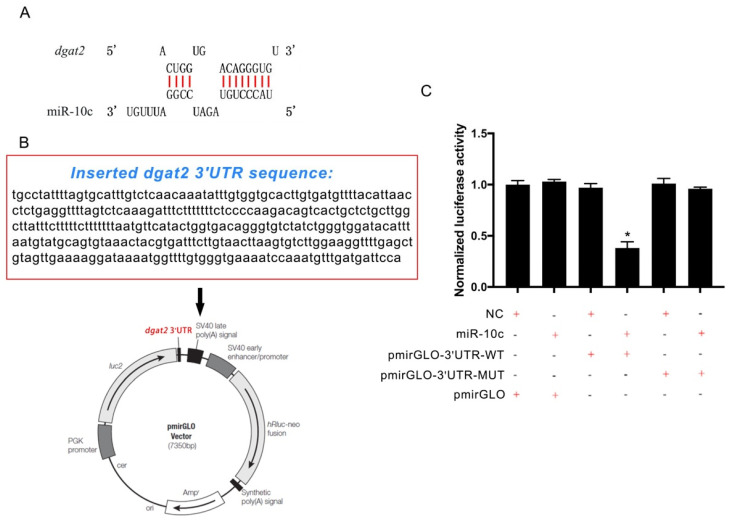
Validation of the miR−10c binding site on *dgat2*. (**A**) Predicted miR-10c binding site within the 3′-untranslated region (UTR) of *dgat2* mRNA, as determined using RNAhybrid; (**B**) Schematic of pmirGLO-*dgat2* 3′UTR plasmid with inserted sequence highlighted in red; (**C**) Dual-luciferase reporter assay results (*n* = 5). * *p* < 0.05.

An RNA hybridization analysis predicted a potential binding site for miR-10c in the 3′UTR of *dgat2* mRNA in GIFT, with a minimum free energy (MFE) of −23.7 kcal/mol (Figure 2A). A fragment of the *dgat2* mRNA 3′UTR containing the putative binding site was cloned into a luciferase reporter plasmid (Figure 2B). The dual luciferase activity assay revealed that only the miR-10c mimic co-transfected with *dgat2*-3′UTR-WT significantly reduced luciferase activity in HEK293T cells (*p* < 0.05), whereas no significant differences in luciferase activity were observed among the other co-transfected groups in HEK293T cells (*p* > 0.05) (Figure 2C). These findings confirm that *dgat2* is a direct target of miR-10c in GIFT.

### 2.3. Regulatory Effect of miR-10c on dgat2 Expression

**Figure 3:**
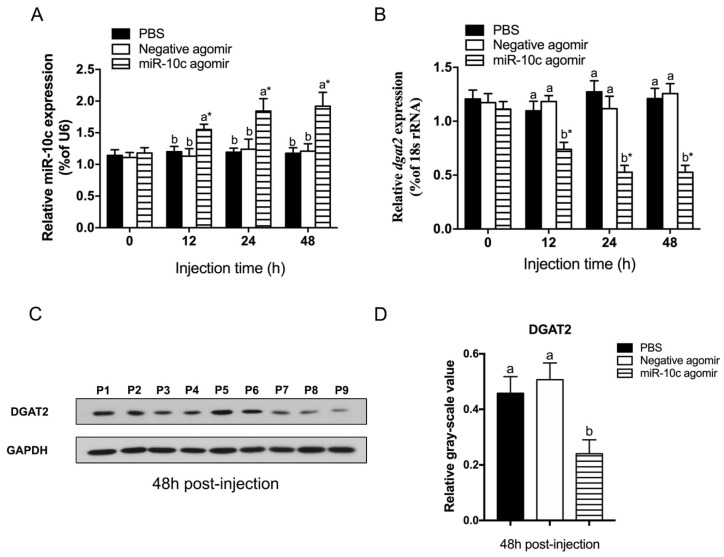
Regulatory impact of miR-10c on *dgat2* expression. Expression profiles of miR-10c (**A**) and *dgat2* mRNA (**B**) in genetically improved farmed tilapia (GIFT) at 48 h post-injection with PBS, negative agomir, or miR-10c agomir (*n* = 9); (**C**) DGAT2 protein levels in fish injected with PBS (P1–P3), negative agomir (P4–P6), and miR-10c agomir (P7–P9) at 48 h post-injection. (**D**) The analysis of gray-scale for DGAT2 protein in fish injected with PBS, negative agomir, and miR-10c agomir at 48 h post-injection. * indicates significant differences between before and after injection or transfection (paired-samples *t* test). Different lowercase letters indicate significant differences among treatments at each sampling point (*p* < 0.05, two-way ANOVA). Notes: The data were analyzed using two-way ANOVA to assess the main effects of different treatments (PBS, negative agomir and micR-10c agomir) and different times (0, 12, 24 and 48 h) and their interaction. Significant interactions were followed by post hoc Duncan’s multiple range test. The significance markers in the figures represent the results of these post hoc comparisons, which were appropriately restricted to comparing groups within the same time point (for treatment effects) or within the same treatment group (for time effects), as such pairwise comparisons are biologically meaningful.

After injection of the miR-10c agomir into the tail vein, the juvenile GIFT showed significantly upregulated hepatic miR-10c expression (*p* < 0.05) (Figure 3A) and downregulation of *dgat2* (*p* < 0.05) (Figure 3B) at 12 h post-injection. Decreased levels of the DGAT2 protein after injection of miR-10c were validated by Western blot analysis (Figure 3C,D). All of these findings show that miR-10c negatively regulates *dgat2* expression.

### 2.4. Effects of dgat2 Knockdown on TG and FFA Contents and Transcript Levels of Lipid Metabolism-Related Genes in Hepatocytes

**Figure 4:**
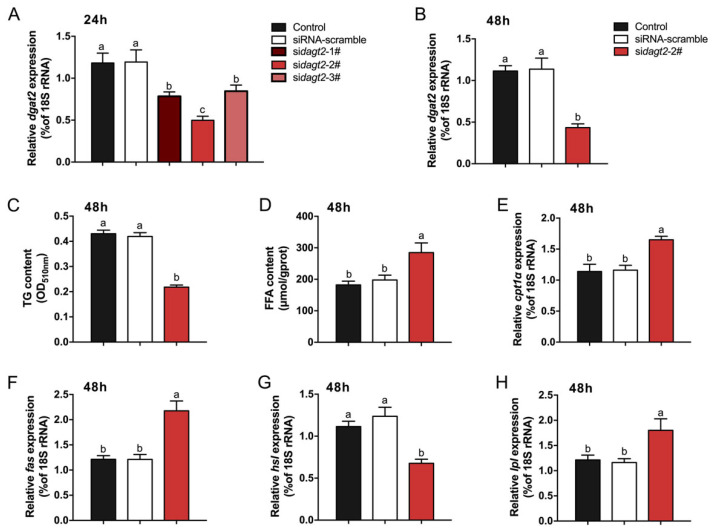
Effect of *dgat2* knockdown on triglyceride (TG) and free fatty acid (FFA) contents and transcript levels of lipid metabolism-related genes in hepatocytes (*n* = 5). (**A**) In vitro si*dgat2* interference for 24 h; (**B**) In vitro si*dgat2*-2# interference for 48 h; (**C**) TG; (**D**) FFA; (**E**) *cpt1α*; (**F**) *fas*; (**G**) *hsl*; (**H**) *lpl*. Different lowercase letters indicate significant differences among treatments at each sampling point (*p* < 0.05, one-way ANOVA).

We designed three *dgat2*-targeting siRNA fragments: si*dgat2*-1#, si*dgat2*-2#, and si*dgat2*-3#. Among these, si*dgat2*-2# exhibited the strongest inhibitory effect on *dgat2* transcript levels in hepatocytes (*p* < 0.05, Figure 4A), so it was used in further experiments. The transcript level of *dgat2* was significantly inhibited (*p* < 0.05) by si*dgat2*-2# at 48 h after transfection (Figure 4B). In addition, knockdown of *dgat2* resulted in significant downregulation of the TG content and *hsl* transcript level (*p* < 0.05, Figure 4C,G) and significant upregulation of the FFA content and transcript levels of *cpt1α*, *fas*, and *lpl* (*p* < 0.05, Figure 4D–F,H).

### 2.5. Establishment of an miR-10c Overexpression Model In Vivo Under Heat Stress

**Figure 5:**
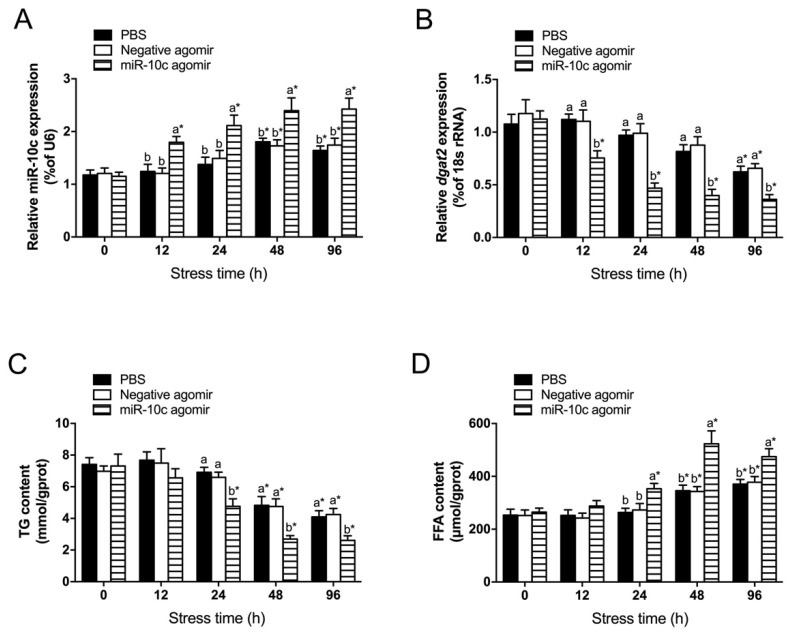
Establishment of a miR-10c overexpression model in vivo under heat stress (*n* = 9). (**A**) hepatic miR-10c; (**B**) hepatic *dgat2*; (**C**) hepatic TG; (**D**) hepatic FFA. * indicates significant differences between before and after injection or transfection (paired-samples t test). Different lowercase letters indicate significant differences among treatments at each sampling point (*p* < 0.05, two-way ANOVA). Notes: The data were analyzed using two-way ANOVA to assess the main effects of different treatments (PBS, negative agomir and micR-10c agomir) and different times (0, 12, 24, 48 and 96 h) and their interaction. Significant interactions were followed by post hoc Duncan’s multiple range test. The significance markers in the figures represent the results of these post hoc comparisons, which were appropriately restricted to comparing groups within the same time point (for treatment effects) or within the same treatment group (for time effects), as such pairwise comparisons are biologically meaningful.

The expression level of miR-10c in hepatocytes was significantly higher (*p* < 0.05) in the miR-10c agomir group than in the PBS or negative agomir groups at 12, 24, 48, and 96 h post-heat stress exposure (Figure 5A). At the same time, hepatic *dgat2* transcript levels were significantly lower (*p* < 0.05) in the miR-10c agomir group than in the PBS or negative agomir groups at 12, 24, 48, and 96 h of heat stress (Figure 5B). Under heat stress, hepatic miR-10c expression levels increased over time (*p* < 0.05) in both the PBS and negative agomir groups, while the transcript levels of *dgat2* decreased (*p* < 0.05).

The hepatic TG content was significantly lower in the miR-10c agomir group than in the PBS and negative agomir groups after 24 h heat stress (*p* < 0.05). The hepatic TG contents in the PBS and negative agomir groups were significantly decreased after 48 h of heat stress, compared with their respective levels at 0 h (Figure 5C). In contrast to the change in hepatic TG content, the FFA content increased under heat stress. The FFA levels became significantly higher (*p* < 0.05) in the miR-10c agomir group than in the PBS and negative agomir groups after 24 h of heat stress. Furthermore, the FFA contents in the PBS and negative agomir groups were significantly higher (*p* < 0.05) at 48 h and 96 h of heat stress, compared with their respective levels at 0 h (Figure 5D).

### 2.6. Effect of miR-10c/dgat2 Axis on Transcript Levels of Lipid Metabolism-Related Genes in the Liver of GIFT Under Heat Stress

**Figure 6:**
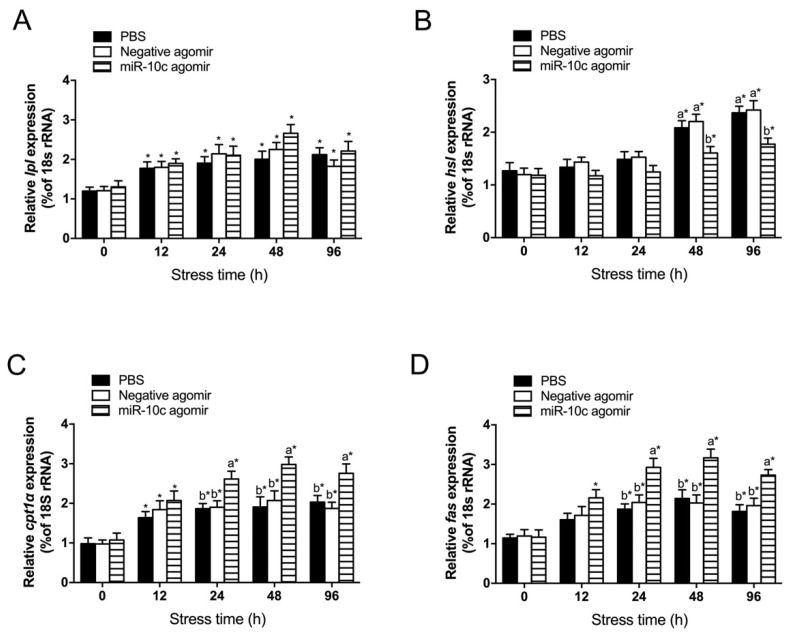
Effect of miR-10c/*dgat2* axis on transcript levels of lipid metabolism-related genes in the liver of GIFT under heat stress (*n* = 9). (**A**) hepatic *lpl*; (**B**) hepatic *hsl*; (**C**) hepatic *cpt1α*; (**D**) hepatic *fas*. * indicates significant differences between before and after injection or transfection (paired-samples *t* test). Different lowercase letters indicate significant differences among treatments at each sampling point (*p* < 0.05, two-way ANOVA). Notes: The data were analyzed using two-way ANOVA to assess the main effects of different treatments (PBS, negative agomir and micR-10c agomir) and different times (0, 12, 24, 48 and 96 h) and their interaction. Significant interactions were followed by post hoc Duncan’s multiple range test. The significance markers in the figures represent the results of these post hoc comparisons, which were appropriately restricted to comparing groups within the same time point (for treatment effects) or within the same treatment group (for time effects), as such pairwise comparisons are biologically meaningful.

During heat stress, the transcript levels of *lpl* were not significantly different among the miR-10c agomir group, the PBS group, and the negative agomir group (*p* > 0.05). However, the *lpl* transcript levels in all three groups were significantly elevated at 12 h of heat stress, compared with those at 0 h (Figure 6A). The *hsl* transcript levels in the liver were significantly lower in the miR-10c agomir group than in the PBS and negative agomir groups at 48 h and 96 h post-stress (*p* < 0.05). However, hepatic *hsl* transcript levels in the miR-10c agomir group were significantly higher at 48 h and 96 h of heat stress than at 0 h (*p* < 0.05, Figure 6B). The transcript levels of hepatic *cpt1α* and *fas* increased over time in the miR-10c agomir group and were significantly higher in the miR-10c agomir group than in the PBS and negative agomir groups at 24 h, 48 h, and 96 h of the heat stress treatment (*p* < 0.05, Figure 6C,D).

## 3. Discussion

Aquaculture is an indispensable source of nutrition globally, but it is threatened by several environmental stressors, particularly thermal stress [1]. High temperatures trigger complex adaptations in energy metabolism to maintain lipid and energy homeostasis. These adaptations involve dynamic changes in TG synthesis and degradation [7,25]. The enzyme DGAT, which catalyzes the final step in TG synthesis, is critically positioned to modulate lipid metabolic responses [11]. However, the molecular regulatory mechanisms of DGAT in heat-stressed fish remain largely unexplored. The results of our study provide novel evidence that miR-10c directly regulates *dgat2* expression in GIFT, highlighting its significant role in regulating lipid metabolism during the heat stress response.

Emerging evidence has demonstrated that miRNAs are crucial regulators of metabolic adaptation in fish under stress conditions, particularly heat stress [20,26]. A series of miRNAs, including miR-34a [27], miR-84a [28], and miR-1 [29], have been shown to modulate stress-induced metabolic reprogramming by directly targeting and inhibiting specific genes. The notable conservation of miR-10c sequences across diverse species highlights its potential role as a critical modulator of lipid metabolism and energy homeostasis under stress conditions [23,30]. Moreover, the significance of miR-10c is acknowledged because of its involvement in diverse biological processes, including development [31], sex differentiation [32] and the immune response [33]. miRNAs typically function by binding to the 3′-UTR of their target mRNAs. In this study, we demonstrated that a miR-10c mimic reduced luciferase activity through binding to the 3′-UTR of *dgat2* mRNA using a heterologous dual-luciferase assay system in human HEK293T cells. While this system is a widely adopted and powerful tool for the initial in vitro screening of miRNA-mRNA binding, we acknowledge that miRNA-mediated repression can be influenced by cell-type and species-specific factors. Therefore, the efficacy of miR-10c observed in HEK293T cells may not be fully representative of its repressive activity in tilapia hepatocytes in vivo. To unequivocally confirm this regulatory axis in a physiologically relevant context, future work should aim to establish a robust piscine hepatocyte model (e.g., a primary tilapia hepatocyte culture or a fish hepatocyte cell line) for functional validation. Furthermore, miR-10c overexpression significantly suppressed *dgat2* transcript levels and DGAT2 protein levels in the liver of GIFT. These results indicate that miR-10c targets and regulates *dgat2* expression at both the transcriptional and post-transcriptional levels in GIFT.

DGAT2 differs from DGAT1 in that it has a single membrane-embedded hairpin and its catalytic domain is located on the cytoplasmic side of the endoplasmic reticulum membrane [34]. The activity of DGAT2 is largely dependent on TG synthesis in mammals and fish [35,36]. To investigate how *dgat2* regulates TG synthesis and lipid homeostasis, we performed siRNA-mediated *dgat2* knockdown in GIFT hepatocytes. Our results show that *dgat2* knockdown stimulated TG hydrolysis while increasing FFA accumulation in GIFT hepatocytes. We hypothesize the existence of a miR-10c-DGAT2-TG pathway that regulates lipid metabolism in GIFT hepatocytes. Gluchowski et al. demonstrated that hepatocyte-specific *DGAT2* inhibition reduced de novo lipogenesis and attenuated hepatic steatosis without exacerbating liver inflammation or fibrosis [37]. In HepG2 cells, a human cell line, inhibition of the MEK/ERK1/2 pathway significantly increased *DGAT2* expression and TG contents [38]. DGAT2 has also been reported to modulate lipid droplet formation in grass carp (*Ctenopharyngodon idella*) kidney cells by regulating TG synthesis [36]. These findings demonstrate that DGAT2 plays a pivotal role in regulating lipid homeostasis in mammals and fish.

Lipid metabolism is a complicated process in fish. We wanted to determine whether a change in the expression levels of *dgat2* affects the expression of other lipid metabolism-related genes in GIFT hepatocytes. Fatty acid synthetase (FAS) is a crucial enzyme for the de novo synthesis of fatty acids, and increases in its expression levels can substantially enhance TG accumulation in vivo [39]. Carnitine palmitoyltransterase-1α (CPT-1α) is the key enzyme for fatty acid entry into mitochondria for β-oxidation [40]. Research on both teleost fish and mammals has indicated that CPT1α and FAS play essential roles in lipid metabolism and the progression of obesity [41,42,43]. In this study, we detected increased *fas* transcript levels in the *dgat2*-knockdown group. This may have enhanced fatty acid synthesis, consequently inducing *cpt1α* expression to promote fatty acid β-oxidation. Lipoprotein lipase (LPL), the rate-limiting enzyme for the hydrolysis of TG, plays a pivotal role in lipid metabolism [44]. In the rainbow trout (*Oncorhynchus mykiss*) adipocyte model, *lpl* expression was found to be closely associated with adipocyte differentiation and the modulation of adipogenesis [45]. In red sea bream (*Pagrus major*), a high-fat diet was found to modulate lipid metabolism via altered *lpl* expression, with elevated *lpl* expression promoting hepatic absorption of dietary lipids [46]. In GIFT hepatocytes, the knockdown of *dgat2* led to increased *lpl* transcript levels, which may have compensated for reduced intracellular TG accumulation by enhancing exogenous lipid uptake. This phenomenon reflects an adaptive response in GIFT hepatocytes, mediated by reprogramming of lipid metabolic pathways under metabolic stress following *dgat2* knockdown. Hormone-sensitive lipase (HSL) plays a crucial role in the lipolysis of stored TGs [47]. In this study, *dgat2* knockdown led to significantly reduced *hsl* transcript levels in hepatocytes, potentially decreasing lipolysis rates and contributing to hepatic lipid homeostasis.

The influence of heat stress on nutrient metabolism in fish is complex. During the initial phase of stress, the glycolysis pathway is activated, resulting in the upregulation of adenosine triphosphate (ATP) synthase and glucose-6-phosphatase, leading to the swift production of ATP to satisfy the increased demand for energy mobilization [48]. With prolonged heat stress, the energy requirements of fish increase, leading to a predominance of lipid metabolism in their energy response [49]. At 48 h of heat stress, the PBS and negative agomir groups exhibited decreased hepatic TG levels but elevated FFA levels. This suggests that lipid metabolism is not prioritized in GIFT during the early stage of the heat stress response, and that TGs start to be mobilized as metabolic damands increase during prolonged exposure. Similar metabolic shifts have been observed in hypoxia-stressed GIFT, where increased lipid utilization compensates for reduced glucose metabolism [50]. Significant changes in the TG and FFA levels in the miR-10c agomir group became evident at 24 h of heat stress, indicating that miR-10c overexpression may accelerate metabolic reprogramming to enhance adaptation to hyperthermia.

We analyzed the impact of miR-10c overexpression on the transcript levels of genes related to lipid metabolism under heat stress. The hepatic *lpl* transcript levels had increased in all treatment groups by 48 h of heat stress. This increase is consistent with prior observations that water temperature and hormonal signals can upregulate LPL, facilitating the increased processing of lipoprotein-derived lipids during the stress response [51,52]. In contrast, hepatic *hsl* was significantly downregulated in the miR-10c agomir group at 48 and 96 h of heat stress, but its transcript levels were still higher than that at 0 h. We speculate miR-10c-mediated DGAT2 expression may initially increase lipolysis before adjusting the balance between TG breakdown and repartitioning. Notably, both *cpt1α* and *fas* were significantly upregulated in the miR-10c agomir group after 24 h of heat stress. CPT1α facilitates β-oxidation of fatty acids, while FAS governs de novo fatty acid synthesis. Thus, this result is indicative of dual enhancement of both catabolic and anabolic pathways. This activation of the dual route suggests that miR-10c*-dgat2* regulates lipid synthesis and oxidation, enabling GIFT to preserve lipid homeostasis even under stressful conditions.

## 4. Material and Methods

### 4.1. Experimental Fish

Heathy GIFT of a similar size were selected for this study. The fish were acquired from the Yangzhong Base of the Freshwater Fisheries Research Center, Chinese Academy of Fishery Sciences. They were collected from the fishing grounds 20 to 30 days post-hatching and subsequently acclimated for a period of 14 days. The average weight of the fish was approximately 5.6 g. During the adaptation period, the fish were distributed across five tanks, each with a capacity of 450 L, with each tank housing 200 individuals. Prior to the experiment, the fish were acclimated for 14 days in a recirculating water system under the following controlled conditions: water temperature at 28 ± 1 °C, dissolved oxygen > 7.0 mg/L, and twice-daily feeding to satiation (08:30 and 17:30) with a standard commercial diet for tilapia obtained from Zhejiang Xinxin Feed Co., Ltd. (Huzhou, China) (33% crude protein, 5% crude lipid). Water temperature, dissolved oxygen, and pH were measured daily using a thermometer, a dissolved oxygen meter, and a pH meter, respectively. Meanwhile, levels of ammonia, nitrite, and nitrate were assessed using a commercial colorimetric test kit (API Freshwater Master Test Kit, Mars, Incorporated, McLean, VA, USA).

All animal experiments in this study were conducted in compliance with the ethical standards set by the Bioethical Committee of the Freshwater Fisheries Research Center (FFRC), Chinese Academy of Fishery Sciences, regarding the care and use of experimental animals (Approval No. 2013863BCE). The human embryonic kidney (HEK293T) cell line used in this study is a commercially available cell line and did not require specific ethical approval for its use.

### 4.2. Bioinformatics Analysis of miR-10c

miR-10c precursor and mature sequence data were downloaded from the miRBase database (MIMAT0042775, http://www.mirbase.org, accessed on 17 April 2025). After eliminating duplicate sequences, bioinformatic analysis was performed. The secondary structure of the miR-10c precursor sequence (oni-miR-10c) was predicted using RNAfold [53]. Conservation analysis of miR-10c was performed using the WebLogo online tool (http://weblogo.berkeley.edu/logo.cgi, accessed on 17 April 2025) [54]. The precursor and mature sequences of miR-10c were aligned using MEGA 11.0 [55], and a phylogenetic tree was constructed using the Neighbor-Joining (NJ) method.

### 4.3. UTR Dual-Luciferase Reporter Assay

A ~300 bp fragment of the GIFT *dgat2* 3′ untranslated region (UTR) (XM_003458972.5), including the seed binding region, was chemically synthesized and cloned into pmirGLO via T4 DNA ligase. The wild-type pmirGLO-*dgat2* 3′UTR vector was amplified after transformation of competent *Escherichia coli* cells. The mutated 3′UTR of *dgat2* (seed binding region 5′-CAGGGU-3′ to 5′-AGUAUC-3′) was generated in accordance with miRNA-target binding principles. The mutant pmirGLO-DGAT2-3′UTR vector was produced in the same way. The 3′UTR sequences were commercially synthesized using all-nucleotide methods by the Wuxi Yixin Biotechnology Co., Ltd. (Wuxi, China). HEK293T cells were co-transfected with 50 nM miR-10c mimic or the negative control (NC), combined with 25 ng of: pmirGLO-*dgat2*-3′UTR-MUT or pmirGLO-*dgat2*-3′UTR-WT or the empty pmirGLO vector. The culture medium for HEK293T cells consists of 90% DMEM (GIBCO, Thermo Fisher Scientific, Grand Island, NY, USA) supplemented with 10% high-quality fetal bovine serum (GIBCO, Thermo Fisher Scientific, USA). Cells are cultured at 37 °C in an atmosphere containing 5% CO_2_. The culture medium was removed by pipette, and the cells were lysed with passive lysis buffer (GIBCO, Thermo Fisher Scientific, USA) at 48 h post-transfection. Firefly luciferase activity was measured by adding 100 µL LAR II substrate to 20 µL supernatant. Renilla luciferase activity was quantified immediately after adding 100 µL Stop & Glo^®^ Reagent (Promega, Madison, WI, USA). The normalized activity was calculated as follows: Firefly luminescence/Renilla luminescence. The miR-10c mimic was commercially synthesized by the RiboBio Co., Ltd. (Guangzhou, China) based on the oni-miR-10c sequence (5′-UACCCUGUAGAUCCGGAUUUGU-3′). The NC was a scrambled miRNA sequence (5′-UUUGUACUACACAAAAGUACUG-3′) with no resemblance to any sequences in the tilapia genome.

### 4.4. Overexpression of miR-10c in GIFT Juveniles

The agomir used in our study was single-stranded RNA, which consisted of 21–23 nt modified as follows: (miR-10c agomir 5′-UACCCUGUAGAUCCGGAUUUGU-3′). The negative agomir had six mismatch mutations from 5′-ACCCUG-3′ to 5′-GAUACU-3′ in the seed region of miR-10c. Both the agomir and the negative agomir were synthesized by RiboBio. All nucleotides were 2′-OMe modified.

A total of 135 GIFT juveniles (5.6 ± 0.3 g) were injected in the tail vein with 1 nmol/g miR-10c agomir, negative agomir, or phosphate-buffered saline (PBS) [56]. Both the miRNA agomirs (overexpression group) and the negative control agomir (NC group) were dissolved in 1 X PBS to ensure identical handling and delivery conditions across all treatment groups. The injection volume was approximately 56 ± 3 µL. The fish were withheld from feeding on the day of the injection. Each treatment group was maintained in triplicate tanks. After injection, the juvenile fish were transferred to nine 600 L culture tanks with the same conditions as those during the acclimation phase. At 0, 12, 24, and 48 h post-injection, three fish per tank were sampled. Following anesthesia with 100 mg/L MS-222, liver tissues were dissected, snap-frozen in liquid nitrogen, and stored at −80 °C until analysis of miR-10c and *dgat2* transcript levels.

### 4.5. dgat2-Knockdown in GIFT Hepatocytes

We designed three siRNA fragments targeting different coding regions of *dgat2* (NCBI accession: XM_003458972.5) of Nile tilapia. A non-targeting scrambled siRNA was constructed as the control (Table S1). The generated siRNA fragments were ligated into the BbsI-digested psiRNA vector, which was then transformed into *E. coli* GT116 cells for storage. The *dgat2*-knockdown plasmid was named psiRNA-*dgat2*, the scrambled plasmid was named psiRNA-NC, and the empty plasmid was named psiRNA(-). Primary tilapia hepatocytes were isolated and cultured in vitro as described previously [57]. Hepatocytes were seeded in standard 12-well tissue culture-treated plates and transfected using Lipofectamine 2000 with psiRNA*-dgat2*, psiRNA-NC, and psiRNA(-) when they reached approximately 80% confluence. The transfected treatment groups were cultured in a constant temperature incubator at 27 °C, 5% CO_2_, and 95% relative humidity. After 48 h of culture, the hepatocytes were harvested for analysis, with five replicates per treatment group.

### 4.6. Heat Stress Treatment of GIFT Juveniles

A total of 180 GIFT juveniles (5.8 ± 0.2 g) were injected in the tail vein with miR-10c agomir, negative agomir, or PBS, as described in Section 2.4. Each treatment group consisted of three replicate tanks. After injection, the juvenile GIFT were distributed among nine 800 L tanks and subjected to heat stress. The water temperature was maintained at 34.5 °C throughout the heat stress period, while all other environmental parameters were the same as those during the acclimation phase. The fish were not fed during the heat stress treatment. At 0, 12, 24, 48 and 96 h of heat stress, three fish per tank were sampled. Following anesthesia with 100 mg/L MS-222, liver tissues were dissected, snap-frozen in liquid nitrogen, and stored at −80 °C for subsequent analysis.

### 4.7. Determination of TG and FFA Contents

The TG content in hepatocytes was determined using the method of Ramirez Zacarias et al. [58]. Briefly, after removing the culture medium (90% DMEM supplemented with 10% high-quality fetal bovine serum) by aspiration, the collected hepatocytes were gently washed three times with PBS, fixed with 10% neutral buffered formalin for 45 min, and then washed three times with PBS. The cells were then stained with Oil Red O for 45 min, rinsed three times with distilled water to remove suspended cells and residual dye, and finally extracted with isopropanol. The absorbance of the extracted solution was measured at 510 nm. The hepatic TG content and free fatty acid (FFA) levels in hepatocytes and liver tissue were quantified using commercial assay kits (Langdun Biological Technology Co., Ltd., Shanghai, China) following the manufacturer’s protocols.

### 4.8. Analysis of miRNA and mRNA Expression by qRT-PCR

The miRNA and mRNA levels were determined in liver cells as described previously [59]. In brief, the samples were using a cell homogenizer for homogenization. miRNAs were extracted using the miRNeasy kit (Takara, Dalian, China) and then reverse-transcribed using the Mir-X^TM^ miRNA First- Strand Synthesis kit (Takara). The miRNAs were quantified using the Mir-X^TM^ miRNA qRT-PCR SYBR^®^ Kit (Takara). For mRNA analysis, total RNA was isolated using TRIzol and cDNA was synthesized using PrimeScript™ RT Master Mix (Takara). Then, qRT-PCR was conducted using SYBR^®^ Premix Ex Taq reagent (Takara) and an ABI QuantStudio5 instrument (ABI, Foster City, CA, USA). All reactions were conducted in triplicate. Relative gene transcript levels were calculated using the 2^−ΔΔCt^ method [60]. The normalization controls were U6 for miRNAs and 18S rRNA for mRNAs. The sequences of primers used for qRT-PCR are listed in Table S2.

### 4.9. Western Blotting

The DGAT2 protein levels were determined by SDS-PAGE followed by Western blot analysis, as previously described [61]. The DGAT2 protein expression levels were rigorously normalized. Specifically, the band intensity of DGAT2 was normalized to the expression level of the housekeeping protein GAPDH within the same sample. To assess DGAT2 protein expression, antibodies against DGAT2 and GAPDH (control) were sourced from HuaAn Biotechnology Co. (Hangzhou, China). The rabbit IgG secondary antibody was obtained from Cell Signaling Technology Inc. (Beverly, MA, USA).

### 4.10. Statistical Analysis

All results are shown as mean ± standard error (mean ± SE). Experimental data were analyzed using SPSS 21.0 statistical software. The data were initially assessed for normal distribution and variance homogeneity. Data from the same experimental group at different sampling times were compared by paired-sample t test [57,59]. Data from different treatment groups at the same sampling time were analyzed by one-way analysis of variance and a post hoc Duncan’s multiple range test. Data from different treatment groups at different time were analyzed by two-way analysis of variance and a post hoc Duncan’s multiple range test. Differences were considered significant at *p* < 0.05.

### 4.11. Hygienic Measures and Disposal

The GIFT was immersed in a 100 mg/L MS-222 anesthetic solution for 5 min. After this period, the fish remained motionless, with its abdomen facing upward and gill movements becoming irregular. At this point, the GIFT was considered to be under deep anesthesia and ready for euthanasia. Subsequently, the fish was euthanized using the rapid cooling method. Specifically, the experimental fish was immersed in a 30 L mixture of ice water at 0 °C (comprising 25 L of ice and 5 L of water) for 10 min, until the gill covers ceased to open or close for at least 60 s. Once euthanasia was confirmed, subsequent sampling procedures were carried out.

## 5. Conclusions

**Figure 7:**
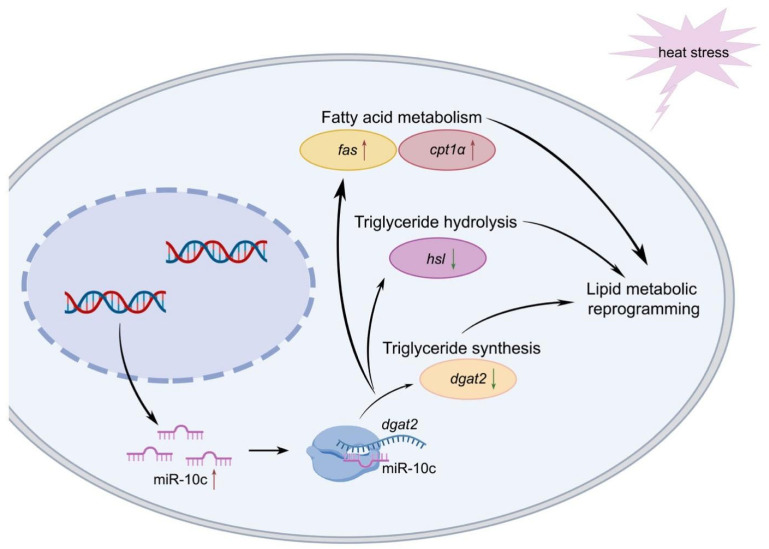
Proposed mechanisms through which miR-10c modulates *dgat2* expression to regulate fatty acid and triglyceride metabolism in GIFT under heat stress. Note: The red and blue arrows respectively indicate the upregulation and downregulation of the genes.

Our findings demonstrate that miR-10c is highly conserved across species. The results of the dual-luciferase assay and miRNA overexpression experiment confirmed that miR-10c directly targets *dgat2* in GIFT. Knockdown of *dgat2* led to upregulation of *cpt1α*, *fas* and *lpl*, and downregulation of *hsl*, indicative of the reprogramming of lipid metabolism in hepatocytes. Under heat stress, miR-10c may promote the degradation of TGs and enhanced fatty acid metabolism via regulation of *dgat2*. This represents an effective adaptation strategy of GIFT to heat stress. Finally, we proposed mechanisms through which miR-10c modulates dgat2 expression to regulate fatty acid and triglyceride metabolism in GIFT under heat stress (Figure 7). Our results not only highlight the critical regulatory role of miR-10c in fish lipid metabolism but also provide valuable insights into the molecular adaptation mechanisms underlying energy homeostasis under heat stress. miR-10c represents a promising target for future strategies aimed at improving the thermal resilience and metabolic health of aquacultured fish species.

## Data Availability

Data is contained within the article or Supplementary Material. Data will be made available on request.

## Supplementary Materials

The following supporting information can be downloaded at https://www.mdpi.com/article/10.3390/ijms26199717/s1.
