# Supplementary material for: miR-10c Targets dgat2 and Affects the Expression of Genes Involved in Fatty Acid and Triglyceride Metabolism in Oreochromis niloticus Under Heat Stress
Source: Int J Mol Sci. 2025 Oct 6;26(19):9717. doi: 10.3390/ijms26199717 (PMC12524535; doi:10.3390/ijms26199717)
Supplement: Supplementary file 1 [file ijms-26-09717-s001.zip › ijms-3818893-supplementary.pdf]

Table S1 *dgat2* siRNA information

	**Sequence ((5´→3´)**
Scramble siRNA	UUCUCCGAACGUGUCACGUTT
Target site1	CATTACTGATCTACATCTT
Target site2	GGAACTCCATCGACTACCT
Target site3	GGTGTTGCCTGCACTGCAT

Table S2 Primer sequences used for qRT-PCR

Primer for qRT-PCR	Primer sequence (5'-3')	GenBank No
miR-10c	TACCCTGTAGATCCGGATTTGT	
dgat2-F	TTCGTTTTCCCATCCTGCGA	XM_003458972.5
dgat2-R	ACAGCATTTCCCGTCCCATT	
lpl-F	CACTGAATGGCTCACCGACT	NM_001279753.1
lpl-R	GTTACCGTCCAGCCGTGTAT	
hsl-F	CGAAAGTTTTGTCCGTGAATA	FJ601660.1
hsl-R	CTGATGCCGCTTGTAGTTTTC	
cpt1α-F	AGAGGCCGTGGACCTATCAT	XM_003440354.4
cpt1α-R	GAGGTGGGGAACACGTACAG	
fas-F	CCAGAATCAGCCTGTGGAGTA	GU433188.1
fas-R	GTTTCAGCCTCAGACTCGTTG	
18S rRNA-F	GGCCGTTCTTAGTTGGTGGA	U67340.1
18S rRNA-R	TTGCTCAATCTCGTGTGGCT	

dgat2: diacylglycerol acyltransferase2; *lpl*: lipoprotein lipase; *hsl*: hormone-sensitive lipase; *cpt1α*: carnitine palmitoyltransferase 1α; *fas*: fatty acid synthase.

Table S3. Alignment of wild-type (WT) and mutant (MUT) sequences surrounding the miRNA seed binding site.

dataset: 1
Target: *dgat2_3UTR*
length: 1231
MiRNA: *miR-10c*
length: 22

mfe: -23.7 kcal/mol
p-value: undefined

Position: 803

```
target 5'      A     UG            U 3'
                   CUGG     ACAGGGUG
                   GGCC     UGUCCCAU
miRNA   3' UGUUUA     UAGA            5'
```

Table S4. Two-way analysis of variance for the effects of different time (0, 12, 24,48 h) and treatment (injection with PBS, negative agomir and miR-10c agomir) on Expression profiles of miR-10c and *dgat2* mRNA of genetically improved farmed tilapia (GIFT).

Relative miR-10c expression			
Source	df	*F*	*p*-value
Treatment	2	17.28748	<0.0001
Time	3	3.61444	0.02768
Treatment × Time	6	1.98287	0.10804
Error	24		
Relative *dgat2* expression			
Source	df	*F*	*p*-value
Treatment	2	41.16744	<0.0001
Time	3	3.29498	0.0377
Treatment × Time	6	4.49002	0.00349
Error	24		

Table S5. Two-way analysis of variance for the effects of different time (0, 12, 24,48 h) and treatment (injection with PBS, negative agomir and miR-10c agomir) on Establishment of a miR-10c overexpression model in vivo under heat stress of genetically improved farmed tilapia (GIFT).

Relative miR-10c expression			
Source	df	*F*	*p*-value
Treatment	2	22.80547	<0.0001
Time	4	18.03067	<0.0001
Treatment × Time	8	1.81567	0.11318
Error	30		
Relative *dgat2* expression			
Source	df	*F*	*p*-value
Treatment	2	30.78427	<0.0001
Time	4	28.1961	<0.0001
Treatment × Time	8	2.41308	0.03834
Error	30		
TG content			
Source	df	*F*	*p*-value
Treatment	2	11.7908	1.66565E-4
Time	4	35.48551	<0.0001
Treatment × Time	8	1.15861	0.35544
Error	30		
FFA content			
Source	df	*F*	*p*-value
Treatment	2	20.71017	<0.0001
Time	4	30.55609	<0.0001
Treatment × Time	8	2.47941	0.03402
Error	30		

Table S6. Two-way analysis of variance examining the effects of different time (0, 12, 24, and 48 h) and treatments (injection with PBS and negative agomir and miR-10c agomir) on the miR-10c/dgat2 axis and its impact on transcript levels of lipid metabolism-related genes in the liver of GIFT under heat stress.

Relative *lpl* expression			
Source	df	*F*	*p*-value
Treatment	2	2.43795	0.10446
Time	4	15.4148	<0.0001
Treatment × Time	8	0.75614	0.64284
Error	30		
Relative *hsl* expression			
Source	df	*F*	*p*-value
Treatment	2	11.21022	2.31371E-4
Time	4	31.98203	<0.0001
Treatment × Time	8	1.16853	0.34974
Error	30		
Relative *cpt1α* expression			
Source	df	*F*	*p*-value
Treatment	2	16.28116	<0.0001
Time	4	23.4385	<0.0001
Treatment × Time	8	1.39715	0.23796
Error	30		
Relative *fas* expression			
Source	df	*F*	*p*-value
Treatment	2	22.65063	<0.0001
Time	4	22.54746	<0.0001
Treatment × Time	8	1.99691	0.0815
Error	30		
